# A novel *TTR* mutation (p.Ala65Val) underlying late-onset hereditary transthyretin (ATTRv) amyloidosis with mixed cardiac and neuropathic phenotype: a case report

**DOI:** 10.1186/s12883-022-02952-3

**Published:** 2022-12-09

**Authors:** Andreas Thimm, Sara Oubari, Julia Hoffmann, Alexander Carpinteiro, Maria Papathanasiou, Peter Luedike, Lukas Kessler, Christoph Rischpler, Christoph Röcken, Isabel Diebold, Tienush Rassaf, Hartmut Schmidt, Christoph Kleinschnitz, Tim Hagenacker

**Affiliations:** 1 Department of Neurology and Center for Translational Neuro- and Behavioral Sciences (C-TNBS), University Hospital Essen, Hufelandstrasse 55, 45147 Essen, Germany; 2 Department of Hematology and Stem Cell Transplantation, University Hospital Essen, Essen, Germany; 3 Department of Cardiology and Vascular Medicine, West German Heart and Vascular Center, University Hospital Essen, Essen, Germany; 4 Institute of Molecular Biology, University of Duisburg-Essen, Essen, Germany; 5 Department of Nuclear Medicine, University Hospital Essen, Essen, Germany; 6 Department of Pathology, Christian-Albrechts-University, Kiel, Germany; 7 MGZ - Medical Genetics Center Munich, Munich, Germany; 8 Department of Gastroenterology and Hepatology, University Hospital Essen, Essen, Germany

**Keywords:** Amyloid cardiomyopathy, Amyloid neuropathy, TTR mutation, Tafamidis, Patisiran, Case report

## Abstract

**Background:**

Hereditary transthyretin (ATTRv) amyloidosis is a rare, genetically heterogeneous and phenotypically variable systemic disease characterized by deposition of misfolded transthyretin fibrils in various tissues. ATTRv cardiomyopathy and progressive axonal polyneuropathy are the most common manifestations, leading to severe disability and ultimately death within approximately ten years. As disease-modifying treatment options evolve, timely diagnosis and treatment initiation are crucial to prevent rapid disease progression.

**Case presentation:**

Here, we report on a 73-year old patient initially diagnosed with cardiac wild-type ATTR (ATTRwt) amyloidosis by endomyocardial biopsy. Molecular genetic analysis revealed a novel *TTR* sequence variant (p.Ala65Val) that is highly likely to be amyloidogenic in light of previously reported *TTR* mutations and the patient’s clinical presentation and family history.

**Conclusions:**

Our findings expand the spectrum of known pathogenic *TTR* mutations and underline the importance of a thorough diagnostic workup in amyloidosis patients including careful genetic testing to avoid misdiagnosis and missing of treatment opportunities and to enable cascade testing and tracking of carriers.

## Background

Hereditary transthyretin (ATTRv) amyloidosis is a rare, life-threatening autosomal-dominant systemic disorder characterized by extracellular deposition of misfolded transthyretin fibrils in various tissues. Severe progressive axonal polyneuropathy (ATTRv-PN) and cardiomyopathy (ATTRv-CM) are the most prominent manifestations. Age at symptom onset ranges from 20 to 80 years. Whilst p.Val50Met is the most common underlying genotype globally and in endemic areas such as certain regions of Northern Portugal and Sweden, in non-endemic areas genetic heterogeneity is high with more than 120 amyloidogenic *TTR* mutations identified to date [1]. There are mutations classically associated with either a predominantly cardiac (p.Val142Ile, p.Ile88Leu) or neuropathic phenotype (p.Val50Met), but both presentations frequently overlap [2, 3]. Genetic heterogeneity and phenotypic variability including varying age at disease onset even in carriers of the same mutation, variable penetrance, insufficient diagnostic workup and low disease awareness frequently lead to a diagnostic delay of several years [1]. As untreated ATTRv amyloidosis leads to death within 10 years on average [4] and highly efficient treatment options for ATTRv-PN emerged in recent years, awareness of typical clinical signs and symptoms is crucial for early diagnosis and treatment initiation. Current treatment options for ATTRv-PN comprise tafamidis, an oral TTR tetramer stabilizer, patisiran, a small interfering RNA, and inotersen, an antisense oligonucleotide, the latter ones both leading to degradation of mutant and wildtype TTR mRNA. Diagnosis of ATTRv amyloidosis relies on sequencing of the whole TTR gene, which is mandatory even in bioptically confirmed cases to distinguish between ATTRv and wildtype ATTR (ATTRwt) amyloidosis because of different treatment approaches. ATTRwt amyloidosis is a frequent cause of heart failure with preserved ejection fraction (HFpEF) occuring almost exclusively in men beyond the age of 60, but usually does not lead to a clinically relevant and progressive peripheral neuropathy. However, the prevalence of ATTRwt amyloidosis, sometimes referred to as senile variant, is far higher than that of ATTRv amyloidosis. In studies of autopsy specimens, cardiac wildtype ATTR deposits can be found in up to 25% of patients aged 80 years or older [5]. Treatment with tafamidis that reduces disease progression is approved for ATTRwt amyloidosis (61 mg daily) as well as ATTRv-CM (61 mg daily) and ATTRv-PN (20 mg daily), while inotersen and patisiran that in some cases even improves functional abilities of affected individuals are only approved for the treatment of patients with ATTRv amyloidosis with peripheral neuropathy [6].

Here, we report on a novel *TTR* mutation underlying late-onset ATTRv amyloidosis with mixed neuropathic and cardiac phenotype initially misdiagnosed as ATTRwt amyloidosis.

## Case presentation

**Fig. 1:**
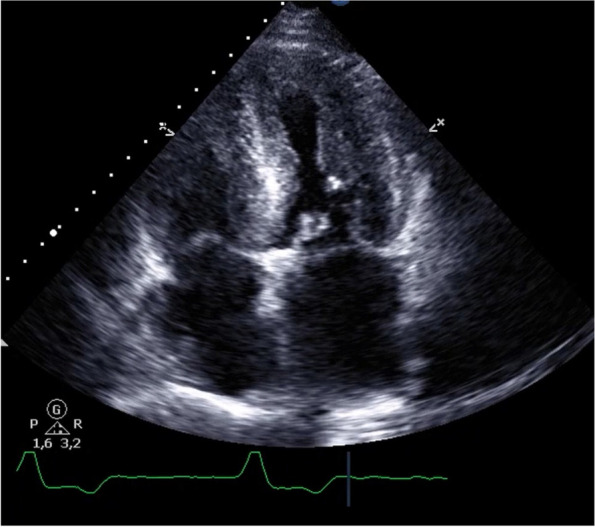
The patient’s echocardiography. Four-chamber view with prominent left-ventricular hypertrophy and granular sparkling typical for amyloidosis

**Fig. 2:**
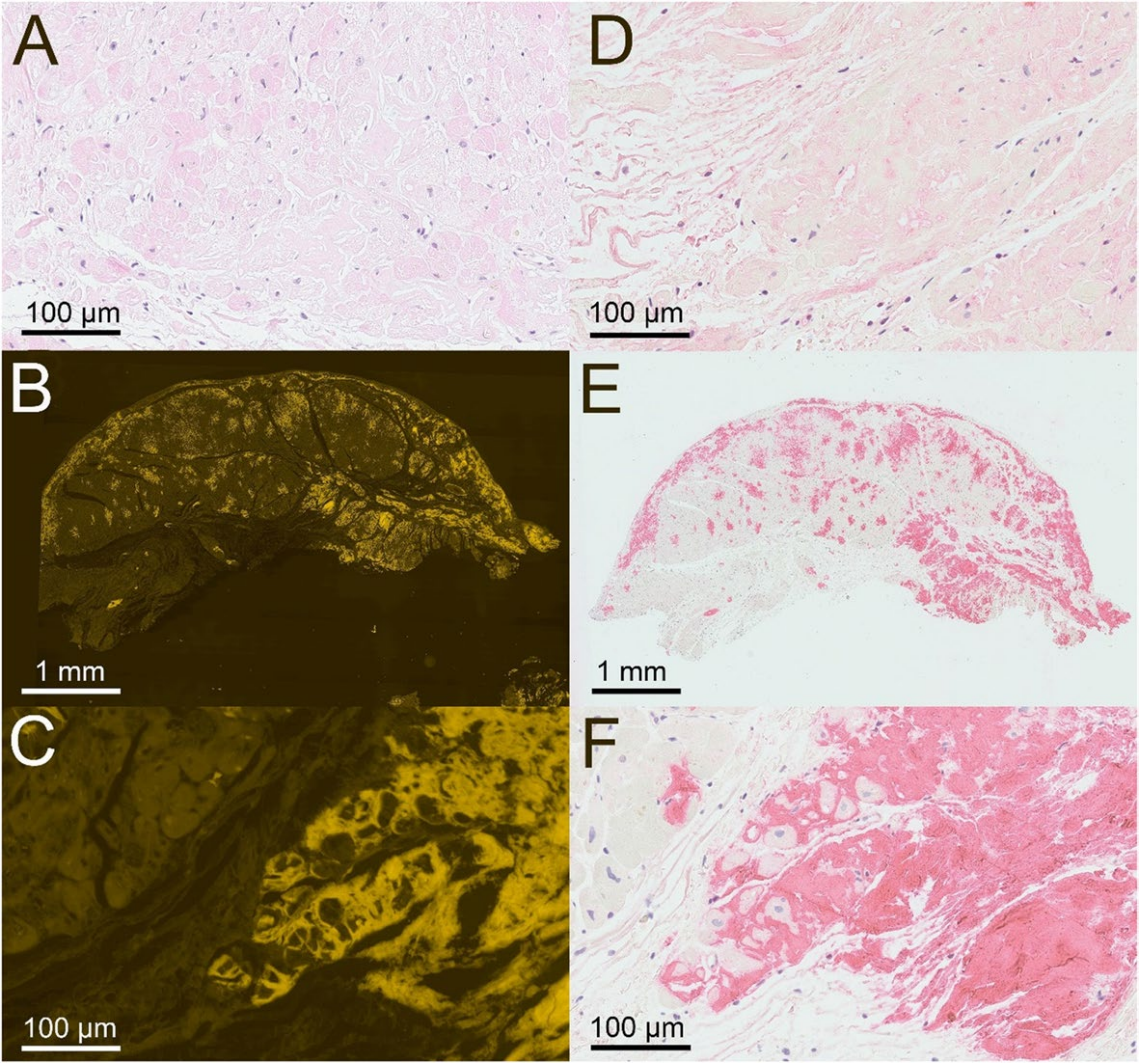
Results of the myocardial biopsy. Hematoxylin and eosin stain (panel **A**, magnification 300x), Congo red fluorescence showing amyloid deposition (panel **B**, magnification 20x, and **C**, magnification 300x), immunohistochemical staining using antibodies directed against immunoglobulin light chains without relevant reaction (panel **D**, magnification 300x), and immunohistochemical staining positive for transthyretin (panel **E**, magnification 20x, and **F**, magnification 300x)

A 73-year old male with progressive impairment of gait, persisting neuropathic pain in his legs, dizziness due to postural changes, and unexplained weight loss of 20 kg (BMI 26.8 kg/m^2^) during the last 2–3 years presented to our outpatient clinic. Three years ago diagnosis of lumbar spinal stenosis was made and decompression surgery was performed to alleviate neuropathic pain. As symptoms progressed, 1 year before the first presentation a second surgical procedure was scheduled and it was only in the preoperative diagnostic workup that echocardiography revealed left ventricular hypertrophy suspected to represent cardiac amyloidosis (see Fig. 1). Endomyocardial biopsy showed extensive amyloid deposition in Congo red stained tissue with a typcial apple green birefringence and fluorescence signal, which could be classified immunohistochemically as TTR derived amyloid (see Fig. 2). Regarding the cardiac manifestation and the patient’s age and sex ATTRwt amyloidosis was suspected and treatment with tafamidis (61 mg daily) was initiated.

**Fig. 3:**
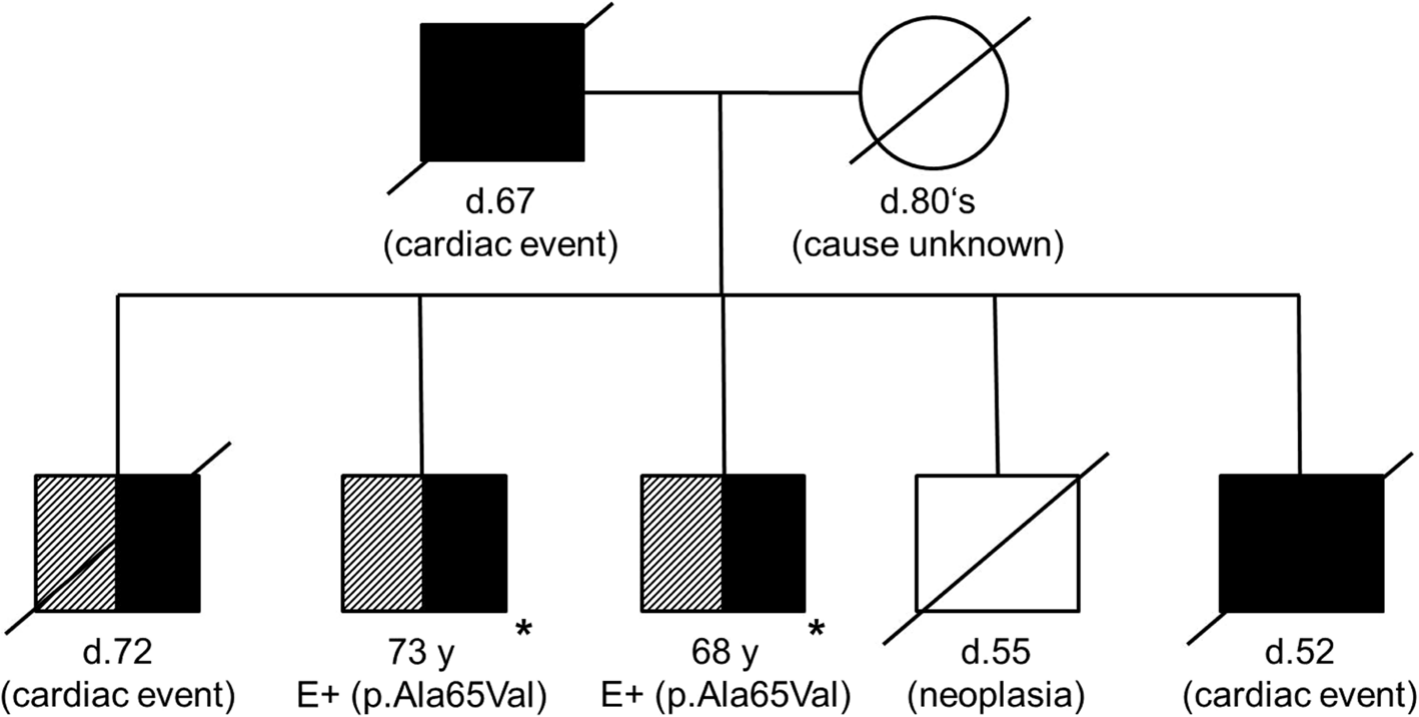
The patient’s pedigree. The reported patient and his only living brother both show cardiomyopathy (hatched) and polyneuropathy (black shading), and the p.Ala65Val variant. One of the three other brothers suffered cardiomyopathy and polyneuropathy. The patient’s father and two of his brothers died from cardiac events. *own documented evaluation, E (Evaluation) = molecular genetic analysis of the *TTR* gene

At the time of referral, the patient was not able to walk unaided for almost 1 year due to weakness of his legs and impairment of balance. Neuropathic pain and dizziness due to orthostatic dysregulation had progressed and the patient reported no obvious improvement after approximately 1 year of treatment with tafamidis. The patient’s family history revealed frequent deaths due to cardiac events in his first-degree relatives (see Fig. 3). His father died at the age of 67 from a cardiac event not further specified, as well as two of the patient’s brothers. One of them reportedly suffered cardiomyopathy and polyneuropathy. The only living brother, aged 68, was considerably impaired by a cardiomyopathy and peripheral neuropathy of unknown etiology.

**Fig. 4:**
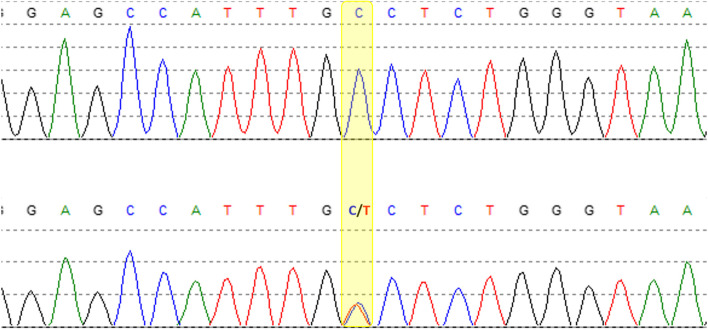
DNA sequencing electropherogram. Reference trace (above) and sample trace (below) with heterozygous C > T point mutation (yellow shaded)

The clinical examination showed a severe impairment of gait due to sensory ataxia, bilateral distal paralyses of the lower extremities (ankle dorsiflexion MRC grade 2/5, plantar flexion MRC grade 2–3/5 on the right side, MRC grade 3–4/5 on the left side). A slight weakness of both hands (MRC grade 4/5) was evident, as well as moderate atrophy of distal limb muscles, distal symmetric hypesthesia, and lost tendon reflexes. Nerve conduction studies (NCS) confirmed a severe axonal sensorimotor neuropathy with bilaterally unobtainable sural and tibial nerve amplitudes. Corneal confocal microscopy (CCM) showed a highly reduced corneal nerve fiber density (7 fibers/mm^2^, reference: > 24 fibers/mm^2^) indicating prominent small fiber involvement. Extensive laboratory testing for potential causes of the neuropathy including HbA1c, vitamin B1, B6, B12, folic acid, TSH, ANA, ANCA, rheumatid factor, hepatitis serology, serum electrophoresis and immunofixation did not yield any relevant abnormalities. There was no history of alcohol abuse or any other exposure to neurotoxic agents. However, molecular genetic analysis (Sanger sequencing, bioinformatic analysis of collected data by means of Mutation Surveyor Version 3.10 and Alamut Visual Version 2.6.1) revealed a heterozygous sequence variant in exon 2 of the *TTR* gene (NM_000371.3 (TTR): c.194C > T, p.Ala65Val, s. Table 1 and Fig. 4), classified as likely pathogenic (class 4) according to the American College of Medical Genetics (ACMG) classification system, which had been reported in ClinVar twice (ClinVar Accession: VCV000448841.4, ClinVar Variation ID: 448841). In ClinVar, the variant is classified as a variant of uncertain significance (two submissions) and once with the condition amyloidogenic transthyretin amyloidosis, but there is no literature on individuals with TTR-related conditions with this genotype. ACMG criteria PM1 (variant located in a mutational hot spot), PM2 (variant absent from general population in databases gnomAD/ExAC), PM5 (novel missense change at an amino acid residue where a different missense change determined to be pathogenic has been seen before), and PP3 (multiple lines of computational evidence supported a deleterious effect on the gene product) were fulfilled [7]. Presuming this genotype to be amyloidogenic, the patient’s brother underwent targeted genetic testing by Sanger sequencing that revealed the same heterozygous sequence variant.

**Table 1:** Snippets of wildtype and mutated amino acid and c.DNA sequences of the TTR gene/protein (mutation highlighted in red)

Mutation on c.DNA level	
Wildtype sequence	GAG CCA TTT GCC TCT GGG AAA
Mutated sequence	GAG CCA TTT GTC TCT GGG AAA
Mutation on amino acid level	
Wildtype sequence	Glu Pro Phe Ala Ser Gly Lys
Mutated sequence	Glu Pro Phe Val Ser Gly Lys

## Discussion and conclusions

Here, we report on a novel point mutation (p.Ala65Val) underlying late-onset ATTRv amyloidosis with a mixed cardiac and neuropathic phenotype, thereby extending the spectrum of known amyloidogenic *TTR* mutations. The amyloidogenic nature of the p.Ala65Val mutation is obvious for several reasons. Firstly, our patient and his brother both show a classical ATTRv amyloidosis phenotype with HFpEF and rapidly progressive peripheral neuropathy with prominent small fiber involvement and autonomic dysfunction leading to orthostatic hypotension, weight loss and neuropathic pain. Lumbar spinal stenosis is common among patients with ATTRv amyloidosis because of amyloid deposition in the ligamentum flavum. Secondly, different amino acid replacements at the same locus have been reported to cause isolated ATTRv-CM (p.Ala65Ser, p.Ala65Thr, p.Ala65Gly) or ATTRv-CM and ATTRv-PN (p.Ala65Asp) previously [8] and in silico analyses via different algorithms relying on evolutionary conservation of sequences (FATHMM) and protein structure/function relationship (SIFT, Sorting Invariant from Tolerated; PolyPhen-2; MutationTaster; Align-GVGD; PROVEAN) predicted a pathogenic effect on amino acid level. Thirdly, the family history of frequent cardiac events without a different common risk factor suggests a genetic background. Although the exact cause of death remained undetermined in the patient’s relatives and no member of the family had been diagnosed with amyloidosis before, unrecognized amyloid cardiomyopathy can be assumed.

We did not perform sural nerve biopsy, since it is not well suited to rule out amyloid neuropathy due to the proximodistal gradient of nerval TTR amyloid deposition [9]. As the patient exhibits a progressive disease course despite treatment with tafamidis, the detection of his *TTR* mutation and evaluation of his severe neuropathy enables a change of the treatment strategy considering patisiran and inotersen as potential new options.

In conclusion, our case report underlines the necessity of careful molecular genetic analysis and history taking in every case of TTR amyloidosis independent from the age at onset, particularly in non-endemic areas, to not overlook rare cases of hereditary TTR amyloidosis and offer appropriate disease-modifying treatments.

## Data Availability

All data analysed are included in this published article.

## Abbreviations

ACMG: American College of Medical Genetics
ANA: Antinuclear antibodies
ANCA: Anti-neutrophil cytoplasmatic antibodies
ATTR: transthyretin-derived amyloid
ATTRv: transthyretin-derived amyloid with underlying *TTR* mutation (v = variant)
ATTRwt: transthyretin-derived amyloid without underlying *TTR* mutation (wt = wildype)
BMI: Body mass index
CM: Cardiomyopathy
ExAC: Exome Aggregation Consortium
gnomAD: Genome Aggregation Database
HFpEF: Heart failure with preserved ejection fraction
MRC: Medical Research Council
NCS: Nerve conduction studies
PN: Polyneuropathy
TSH: Thyroid-stimulating hormone
TTR: Transthyretin
